# Exploiting B Cell Receptor Analyses to Inform on HIV-1 Vaccination Strategies

**DOI:** 10.3390/vaccines8010013

**Published:** 2020-01-01

**Authors:** Christoph Kreer, Henning Gruell, Thierry Mora, Aleksandra M. Walczak, Florian Klein

**Affiliations:** 1 Laboratory of Experimental Immunology, Institute of Virology, Faculty of Medicine and University Hospital Cologne, University of Cologne, 50931 Cologne, Germany; christoph.kreer@uk-koeln.de (C.K.); henning.gruell@uk-koeln.de (H.G.); 2 German Center for Infection Research, Partner Site Bonn-Cologne, 50931 Cologne, Germany; 3 Laboratoire de Physique de l’École Normale Supérieure (PSL University), CNRS, Sorbonne Université, Université de Paris, 75005 Paris, France; tmora@phys.ens.fr (T.M.); awalczak@lpt.ens.fr (A.M.W.); 4 Center for Molecular Medicine Cologne (CMMC), University of Cologne, 50931 Cologne, Germany

**Keywords:** B lymphocytes, B cell receptors, antibodies, antibody repertoire, immunoglobulin, Rep-seq, Ig-seq, vaccination strategy, HIV-1, broadly neutralizing antibodies

## Abstract

The human antibody repertoire is generated by the recombination of different gene segments as well as by processes of somatic mutation. Together these mechanisms result in a tremendous diversity of antibodies that are able to combat various pathogens including viruses and bacteria, or malignant cells. In this review, we summarize the opportunities and challenges that are associated with the analyses of the B cell receptor repertoire and the antigen-specific B cell response. We will discuss how recent advances have increased our understanding of the antibody response and how repertoire analyses can be exploited to inform on vaccine strategies, particularly against HIV-1.

## 1. Introduction

In order to protect from a vast number of different pathogens, human B cells are able to generate a remarkable diversity of different B cell receptors (BCRs). During B cell development and maturation, these receptors are built by recombination and mutation processes resulting in a virtually unlimited number of different antibodies (i.e., soluble BCRs). However, certain pathogens, such as HIV-1, challenge the immune system by the ability to rapidly escape from immune pressure [1,2], resulting in an ongoing adaptation of the immune response against these pathogens. This evolutionary arms race between pathogens and the immune system leaves footprints in our immunological memory that can describe developmental pathways towards an adapted immune response. Deciphering these pathways holds the potential to greatly improve our understanding of critical steps in lymphocyte receptor development and may inform on novel vaccination strategies. For a long time, experimental setups and bioinformatics pipelines were limited in assessing the diversity of lymphocyte receptor repertoires or in identifying subtle immunological imprints after infections or vaccinations. The advent of advanced single cell cloning and next generation sequencing (NGS) methods has revolutionized the field and opened the door to investigate adaptive immune receptor repertoires (AIRR) at an unprecedented depth. In this review, we summarize recent developments that have fostered our understanding of B cell biology and antibody responses. Focusing on the development of highly potent broadly neutralizing antibodies against HIV-1, we discuss how a detailed knowledge of the human B cell repertoire may support the development of novel vaccination strategies.

## 2. B Cell Receptor Diversity

**Figure 1:**
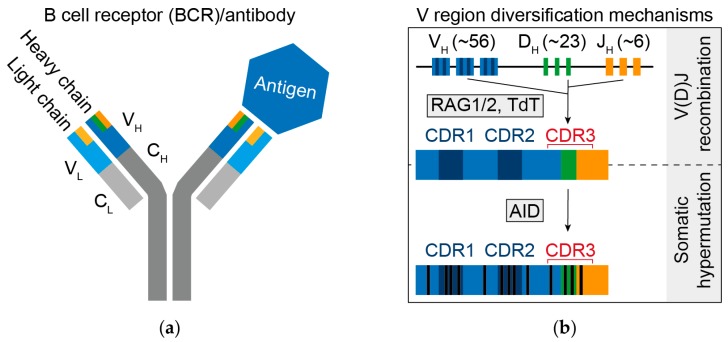
Structure and generation of B cell receptors (BCRs)/antibodies. (**a**) Schematic representation of an antibody. BCRs are composed of heavy (H) and light (L) chains, which can be separated into variable (V) and constant (C) regions. Heavy and light chain variable regions make contact with the antigen. Light chain constant regions come in two different isotypes (kappa and lambda) and heavy chain constant regions in five (IgM, IgD, IgG1-4, IgA1-2, and IgE; not depicted). (**b**) V region diversification mechanisms. V(D)J recombination forms the CDR3s of the naïve B cell receptors. During the process of affinity maturation, somatic hypermutation mediated by activation-induced deaminase (AID) results in the development of mutations within B cell receptors/antibodies. RAG1/2: Recombination-activating gene 1/2, TdT: terminal deoxynucleotidyl transferase, CDR1/2/3: complementarity determining region 1/2/3.

Antibodies are composed of heavy and light chains, both of which are divided into a constant and a variable region (Figure 1a). The different isotypes for the heavy chain constant regions mediate different effector functions and are grouped into the classes IgM, IgD, IgG1-4, IgA1-2, and IgE [3]. The variable regions of heavy and light chains form the paratope that contacts the epitope on a particular antigen (e.g., on a bacterial or viral surface protein). Two essential steps act during the lifespan of a B cell to generate B cell receptor diversity: (i) the V(D)J recombination process that builds the naïve (i.e., antigen-inexperienced) B cell repertoire, and (ii) somatic hypermutation (SHM) during the process of affinity maturation that generates high affinity B cell receptors and antibodies (i.e., the antigen-experienced repertoire).

The initial diversity of the B cell repertoire results from the assembly of the B cell receptor during early B cell development in the bone marrow. The recombination-activating gene (RAG) 1/2 enzymes recombine variable (V), diversity (D), and joining (J) gene segments of the immunoglobulin heavy (IgH) chain locus to first assemble the heavy chain variable region, followed by V and J gene segment recombination within the Ig kappa (IgK) and Ig lambda (IgL) loci [4]. Junctional diversity is further increased by RAG1/2 and other enzymes through the generation of palindromic (P) nucleotides, as well as by the terminal deoxynuclotidyl transferase (TdT) through the addition of non-template (N) nucleotides [5] (Figure 1b). Heavy and light chain V genes exclusively encode for two complementarity determining regions (CDR1 and CDR2) that are usually structurally exposed at the tip of the antibody and contribute to antigen recognition. A third CDR (CDR3) is generated by the V(D)J recombination process and is the most variable part within B cell receptors and antibodies. The CDRs are interspersed and flanked with framework regions (FWR) that mainly function as a scaffold for the overall immunoglobulin (Ig) fold. However, mutations in FWRs have been shown to also influence binding affinity as well as neutralizing activity [6,7].

There are currently at least 56 functional V, 23 D, and 6 J genes described for the IgH locus [8], which results in a theoretical combinatorial number of 7728 different heavy chain variable regions. The IgK locus can recombine to 205 different kappa light chain variable regions from 41 V and 5 J genes, whereas the IgL locus might recombine up to 165 different lambda light chains from 33 V and 5 J genes [8]. Combinatorial pairing of heavy and light chains yields a theoretical diversity of about 2.9 × 10^6^ different antibodies. Including the P/N nucleotides, the theoretical number of different antibody sequences is vastly higher than the total number of estimated B cells in the human body (10^12^) [9]. However, all those antibody sequences are not equally likely to be generated. Their generation probability spans 30 orders of magnitude for IgH alone [9,10], with additional diversity being generated by insertions and deletions [11]. Of note, due to sampling issues, the number of B cell clones whose size falls below the detection threshold is unknown, rendering estimates of total B cell counts unreliable [12,13].

Naïve B cells circulate between secondary lymphoid tissues (e.g., lymph nodes and spleen) until they recognize their cognate antigen [14]. Upon antigen contact, B cells can be recruited to lymphatic structures called germinal centers present in secondary lymphoid tissues. There, the recognition event is able to trigger a second step of diversification called affinity maturation. Affinity maturation is mediated by the enzyme activation-induced deaminase (AID) as well as B cell expansion and selection [15]. AID introduces SHM including substitutions, insertions, and deletions into the variable regions, generating possible progenies that express B cell receptors that get selected for higher antigen affinity (Figure 1b) [15,16]. SHM is favored but not limited to hot spot motifs and multiple SHM hotspots have been identified [17,18]. In addition to their context preference, SHMs tend to occur close to each other along the sequence [10]. They are typically more pronounced in the CDRs than in the framework regions due to positive selection as well as higher frequency of AID motifs [18]. Including SHM into the calculation of potential BCRs results in an almost infinite number of different receptors. Finally, AID activity is able to mediate heavy chain class switch from IgM/IgD to IgG, IgA, or IgE. Different B cell subtypes and antibody classes are of critical importance and their functions have been reviewed elsewhere [3,14,19].

## 3. Challenges and Advances in B Cell Receptor Analyses

**Figure 2:**
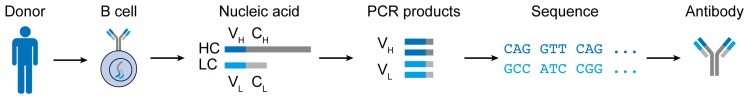
Analyzing B cell receptors from human donors. B cells are isolated, and DNA or RNA is extracted and subjected to B cell receptor sequence amplification. Amplicons are sequenced and can be used for recombinant antibody production. HC: heavy chain, LC: light chain, V: variable region, C: constant region, V_H_: heavy chain variable region, V_L_: light chain variable region.

Dissecting the humoral immune response is a challenging task. Analyses of antibodies on the serum level, for example by ELISA, affinity chromatography, or mass spectrometry [20], are usually limited to characterizing the polyclonal antibody response. Genetic B cell analyses, on the other side, facilitate single cell (i.e., single antibody) resolution. To this end, antibody-coding nucleic acids (DNA or RNA) are extracted from B cells, amplified, and sequenced (Figure 2). Importantly, complete sequences of matched heavy and light chains allow for recombinant production of antibodies and thus allow studying antibody functions on a monoclonal level (Figure 2). In this section, we describe the challenges arising at the different steps of genetic BCR analyses and discuss advantages and disadvantages of individual strategies.

### 3.1. Subset Identification

**Figure 3:**
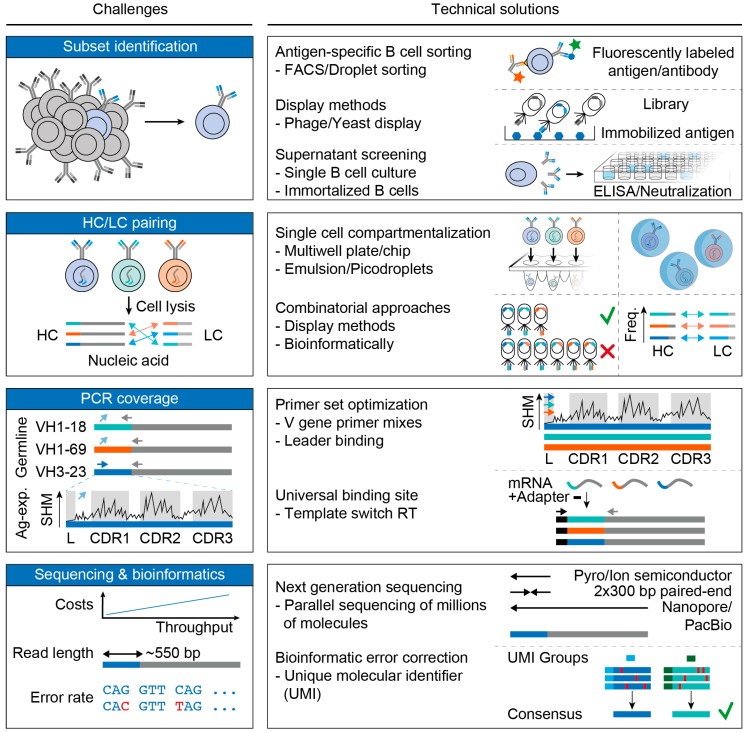
Technical solutions to challenges for B cell receptor analyses. HC: heavy chain, LC: light chain, Freq: frequency, Ag-exp: antigen-experienced, SHM: somatic hypermutation, VH: V gene segment of the heavy chain, L: leader region, CDR1/2/3: complementarity determining region 1/2/3, mRNA: messenger RNA.

B cells can be subdivided into different subsets. These comprise (i) B cells at different developmental stages (e.g., pro-B cells, immature B cells, mature B cells), (ii) antigen-naive and antigen-experienced B cells, (iii) functional subsets, such as regulatory, effector, or memory B cells, or iv.) B cells with defined specificity (e.g., HIV-1_Env_-reactive). Depending on the scientific question, it is often required to analyze an individual B cell subset and identification of such subsets can take place at distinct steps of an experimental pipeline (Figure 3, first row).

Different B cell subsets can be enriched or isolated by sorting techniques such as magnetic-activated cell sorting (MACS) or fluorescence-activated cell sorting (FACS) [21,22]. Importantly, FACS allows collecting and further processing target cells either in bulk approaches or as single cells in multi-well plates. Recently, novel microencapsulation systems have been used to encapsulate single B cells into picoliter droplets [23,24]. The combination of single cell encapsulation with fluorescence-activated sorting (fluorescence-activated droplet sorting (FADS), reviewed in [25]) allows processing of single antigen-specific B cells in compartments that are a million times smaller than the wells of multi-well plates, which significantly increases the throughput capacity.

In order to isolate antigen-specific B cells, one of the following approaches (reviewed in [26,27]) can be applied (Figure 3, first row): (i) Antigen-derived baits that are fluorescently labeled can be used to identify and sort antigen-reactive B cells. This can be achieved by fluorescently-labeled proteins [28,29,30,31,32,33,34], antigens presented on virus-like particles [35,36] or cells [37], or by pathogens themselves [38]. (ii) Antibody libraries that are expressed on phages or yeast cells can be selected for binding to antigen-derived proteins or whole pathogens [39,40]. Of note, however, the random pairing of heavy and light chains in combinatorial libraries does not allow to infer a representative picture of the underlying antibody response. (iii) Single B or plasma cells or immortalized B cells can be expanded and stimulated to secrete antibodies that can be tested for antigen-binding or neutralizing activity [41,42,43,44]. Importantly, recombinant proteins and other baits that are used for selecting antigen-specific antibodies can critically differ in their structure and glycosylation pattern from their native counterpart. Indeed, the generation of optimized bait proteins [30] or native-like envelope trimers [45] were critical steps to improve the isolation of potent HIV-1 broadly neutralizing antibodies (bNAbs) by antigen-specific sorting strategies [31,46,47,48]. Very recently, a combination of single cell co-encapsulation and DNA-tagged recombinant proteins has even been used to directly map antibody sequences to their antigen specificity [49].

### 3.2. Pairing of Heavy and Light Chains

Direct heavy or light chain RT-PCR and sequence analyses from bulk-sorted B cells allow to infer B cell repertoire characteristics such as clonal distributions, V(D)J recombination, and somatic hypermutation [50,51,52,53,54,55]. However, the native pairing information of heavy and light chains is essential to fully describe an individual antibody, e.g., for recombinant expression. Although pairing information can be restored to some extent from bulk analyses (i.e., by bioinformatic approaches) [56], the most robust way to achieve these information is either by single cell sorting into multi-well plates [57,58,59,60] or by co-encapsulation of single cells and RNA-capture or barcode beads (e.g., with the 10× Genomics chromium system) in picoliter droplets [23,24,61,62,63,64,65] (Figure 3, second row). Single cell sorting into multiwell plates is typically limited in throughput to tens of thousands of cells, whereas encapsulation systems allow throughputs of hundreds of thousands of cells. However, droplet occupancy follows a Poisson distribution and requires limiting dilutions of the sample and beads [61]. As a consequence, the majority of all droplets remains empty or contains only unpaired cells or beads, which can lead to a high loss of input material. Alternatives for heavy and light chain paring comprise combinatorial yeast or phage display libraries that can be screened for reactivity (reviewed in [66]). However, due to their stochastic nature, such display approaches also contain artificial heavy and light chain pairs.

### 3.3. PCR Coverage

All current approaches require an initial PCR-based amplification of the BCR-encoding DNA or mRNA/cDNA. The diversity of the B cell repertoire poses distinct challenges to this amplification step: (i) All possible V gene segments need to be covered by the PCR and (ii) priming sites may have been somatically hypermutated and are therefore prone to decreased amplification efficiencies (Figure 3, third row). PCR amplification of antibody heavy and light chains has thus been performed with V gene-specific multiplex primer mixes [57,58,67,68,69,70,71]. The majority of these primers were designed against the 5′ end of the coding region of the V gene, which is sufficient for amplifying most antibody sequences. However, HIV-1 bNAbs, for instance, have been shown to accumulate high levels of SHM as well as insertions and deletions [7,30,72,73]. In order to increase priming efficiency, primer mixes have been designed against the 5′ end of the less-mutated leader region that encodes the antibody secretion peptide. These mixes have been demonstrated to be superior for the isolation of highly mutated HIV-1-reactive antibodies [72,74].

Whereas primer sets perform well in single cell cloning approaches, they may introduce primer biases in bulk PCR amplification approaches [75]. This can pose a critical disadvantage. A method that is able to overcome this limitation is the rapid amplification of 5′ cDNA ends (5′RACE) [76], which has been adapted to bulk [77] and single cell approaches [60,78]. Commonly applied protocols include template-switching (TS) reverse transcription, which introduces a TS-oligo during cDNA synthesis. The TS-oligo bears a universal priming site that can be used together with a constant region reverse primer to amplify any antibody variable region independent of the incorporated V gene segment.

### 3.4. Sequencing and Bioinformatics

Sequence analysis of amplified heavy and light chains can be demanding in several aspects (Figure 3, fourth row). First, depending on the B cell subset of interest, the required throughput can vary from a few hundred to millions of antibody sequences. Second, the sequencing method needs to reliably cover the whole region of interest (variable region ~500–600 bp). Third, SHM needs to be distinguishable from sequencing errors, thus requiring low sequencing error rates or error-correction techniques.

Classical Sanger sequencing is frequently employed in the analyses of B cell receptor subsets such as antigen-specific memory B cells in the blood [34,79,80]. Due to the inability to sequence bulk-amplified heavy and light chains, next generation sequencing (NGS) techniques such as pyro-, ion-semiconductor-, or illumina dye sequencing are typically preferred over sanger sequencing for high-throughput analyses [50,51,52,53,54,55]. However, they often suffer from shorter read lengths and higher error rates. Molecular barcoding techniques and bioinformatics pipelines have been developed to account for both PCR- and sequencing-induced errors [81]. To this end, unique molecular identifiers (UMI) are introduced during cDNA generation by template-switching reverse transcription. Moreover, protocols for long read parallel sequencing (e.g., SMRT and Nanopore sequencing) have been recently applied to analyze BCR sequences [63,82].

Finally, high-throughput analyses of millions of different antibody sequences require advanced bioinformatics pipelines. Several bioinformatics tools have been reported [83] and standardized protocols on reporting antibody sequences have been developed by the Adaptive Immune Receptor Repertoire (AIRR) Community [84,85]. A detailed description of the methods applied is beyond the scope of this review and they are covered in previous reviews [83,86].

## 4. Informing about Vaccination Strategies (I): Molecular Characterization of Broadly HIV-1 Neutralizing Antibodies

Only a small fraction of HIV-1-infected individuals develop highly potent bNAbs and detailed analyses of B cell receptors and antibodies at a single cell level have been limited to a few dozen subjects. Nevertheless, these critical investigations have revealed sequence and structural characteristics of potent HIV-1 neutralizing antibodies that were repeatedly observed across different individuals. These features can include high levels of somatic hypermutation, the presence of unusual insertions or deletions, and/or long heavy chain CDR3 (CDRH3) regions. Thus, vaccine-mediated HIV-1 bNAb induction may require specifically tailored strategies for B cell activation and maturation.

Isolated highly potent bNAbs can serve as templates for the development of such strategies. Over the last decade, numerous bNAbs have been identified by bait-specific single cell sorts or B cell microcultures. Most of these antibodies were obtained from the memory B cell pool as well as, occasionally, from plasma cells [72,87,88]. This suggests that both, memory and antigen-secreting B cells, can in principle serve as a valuable source for bNAb isolation and characterization. All HIV-1 bNAbs target epitopes on the HIV-1 envelope protein (Env) that include the CD4 binding site (CD4bs), glycan-dependent targets on the variable Env loops (V1/V2, V3), the fusion peptide and the membrane-proximal external region (MPER) of gp41, and sites spanning the gp120 and gp41 subunits [27,89].

In clinical trials, bNAbs have been shown to suppress viremia and delay the time to viral rebound after interruption of antiretroviral therapy (ART) [90,91,92,93,94,95,96]. Of most relevance for HIV-1 vaccine efforts, however, bNAbs are highly effective in preventing infection in animal models [97,98,99,100]. As proof-of-concept trials for passive immunization using the CD4bs bNAb VRC01 in humans are ongoing (ClinicalTrials.gov: NCT02716675, NCT02568215), it is widely believed that induction of potent bNAbs by vaccination will confer protection from HIV-1.

Among the highly potent HIV-1 bNAbs, antibodies of the VRC01- and 8ANC131-classes target the CD4 binding site (CD4bs) on the HIV-1 Env. They are particularly noteworthy for their restricted V gene usage [30,72,101] facilitating the VH1-2 or VH1-46 gene segments. Importantly, members of the potent VRC01-class of bNAbs have now been identified in at least 12 individuals, demonstrating their capacity to be reproducibly induced [30,72,88,101,102,103,104,105,106]. Such a reproducible development of very similar antibodies in different individuals is often referred to as convergent or stereotypical antibody responses or described as “public antibodies”. Identifying convergent immune responses is informative for vaccine design because strategies that induce such a repeatedly observed type of immune reaction may be broadly applicable on a population-level. Indeed, B cell analyses have revealed convergent V gene responses not only against HIV-1 but several pathogens after infection and/or vaccination (Table 1).

VRC01-class CD4bs bNAbs demonstrate the same mode of CD4bs recognition that is dominated by the CDR2 of the heavy chain (CDRH2) [101]. To avoid steric clashes, they share an additional restriction for use of an unusually short (five amino acids) light chain CDR3 (CDRL3), which is found in only ~1% of antibodies [102,128]. Finally, they show extensive levels of somatic hypermutation of up to >30% on the nucleotide level (i.e., >100 mutations) from their inferred antibody germline sequences [30,72,102,104].

Members of HIV-1 bNAb classes targeting other epitopes are generally less restricted in terms of their V gene usage but often share other sequence and structural characteristics. For example, bNAbs binding to the V1/V2 apex region typically carry CDRH3s of extraordinary length that are required to penetrate the extensive Env glycan shield [129,130]. Compared to the average CDRH3 lengths of approximately 15 amino acids in the naïve and memory B cell receptor repertoires [131], V1/V2-targeting bNAbs have been identified that have >2-fold longer CDRH3s (e.g., VRC26.25 and PGDM1400 with CDRH3 lengths of ≥34 aa [31,48]). Similarly, relatively long CDRH3s are also found in HIV-1 bNAbs targeting other glycan-related epitopes (e.g., V3 loop, gp120/gp41 interface) or the gp41 MPER [89]. In addition, some bNAbs display poly- and/or autoreactivity [132,133], features that are often associated with long CDRH3s and are generally counterselected during B cell maturation [57]. Overall, the consistent observation of one or multiple rare features in potent HIV-1 bNAbs highlights some of the difficulties for their induction through vaccination. However, several antibodies with considerable breadth and potency but lower levels of somatic hypermutation and more regular CDRH3 lengths have now been identified [36,105,134,135]. These antibodies may be more readily inducible and serve as blueprints for facilitating vaccine strategies.

## 5. Informing about Vaccination Strategies (II): B Cell Receptor Repertoire Analyses

Due to the unusual sequence and structural characteristics of most highly potent HIV-1 bNAbs, unconventional approaches to vaccination are likely to be required. Strategies that have been proposed include epitope-based and antibody lineage-based vaccine designs [136]. Epitope-based vaccination strategies use immunogens that mimic the general structure of vulnerable Env sites, in principle allowing for the development of multiple bNAb classes against the same target region. However, when reverted to their inferred germline sequence, many HIV-1 bNAbs show considerably reduced or fully abrogated binding to HIV-1 Env [72,137,138,139]. To this end, antibody-lineage based vaccination strategies employ designed immunogens that interact with inferred unmutated bNAb precursors to initiate the development of a particular bNAb lineage [140]. Although bNAb precursor cell frequency in the repertoire is only one of a number of factors that will determine the potential success of lineage-based vaccine design, a comprehensive understanding of the composition of the B cell receptor repertoire in healthy individuals can provide critical information to guide the development of vaccination pathways [141].

As seen for the majority of antibodies, most potent HIV-1 bNAbs are strongly dependent on interactions mediated by the CDR3 of the heavy chain. Compared to the distribution in the overall memory B cell repertoire, many HIV-1 bNAbs have relatively long CDRH3s which have been suggested to be largely generated during VDJ recombination [142]. Particularly long CDRH3s are required for many bNAbs targeting the V1/V2 apex region of Env. Notably, among näive B cell repertoires of healthy individuals, CDRH3 lengths of 28 amino acids and more have been identified in less than 0.5% of sequences [142], and a CDRH3 length of 30 amino acids as seen for bNAb PG9 was found to be exceedingly rare (0.01%) [143]. Although this suggests difficulties for CDRH3-based HIV-1 vaccination, the potential contribution of BCR repertoire analyses to vaccine design was recently demonstrated when precursor frequencies of the CDRH3-dominated bNAb BG18 [144,145] were determined to inform on the selection of an immunogen targeting BG18-like precursors [146]. Among 1×10^9^ CDRH3 sequences from a total of 14 healthy donors, BG18-like sequences were identified in all individuals [55,146]. Importantly, rare immunogen-reactive B cells could subsequently be isolated from additional healthy donors [146]. Moreover, antibody lineage-based vaccination strategies that aim to engage precursors of the VRC01-class of CD4bs bNAbs have entered the clinical stage with the germline-targeting immunogen eOD-GT8 (ClinicalTrials.gov: NCT03547245) [147]. As mentioned before, antibodies of this class are particularly restricted for usage of the VH1-2*02 allele and a 5 amino acid CDRL3 [101]. BCR repertoire analyses revealed that potential VRC01-class precursor B cells are exceptionally rare [147,148,149]. In addition, allelic variation can result in the lack of naïve B cells derived from the key VH1-2*02 allele [149]. Nevertheless, eOD-GT8-reactive naïve B cells could be identified in a majority of HIV-1-negative donors (14/18) [147,148], providing repertoire analysis-based support for advancing the eOD-GT8 immunogen to be evaluated in a clinical setting.

While germline-targeting immunogens are designed to initiate a particular B cell lineage, subsequent immunizations with additional antigens will likely be required to induce the development of broad and potent mature antibodies through additional rounds of affinity maturation [140]. To this end, interrogations of the natural development of bNAbs in HIV-1 elite neutralizers may be highly informative for immunogen design. High-throughput parallel sequencing methods of the B cell receptor repertoire combined with bioinformatical processing and phylogenetic analyses have facilitated to reconstruct the inferred development of antibody lineages [56,102,103,104,105,135,139,150,151,152,153,154,155,156,157,158]. Of note, when informed by template sequences of bNAbs obtained through single cell approaches, high-throughput BCR sequencing methods can identify antibodies with higher breadth and potency [159]. Of particular relevance for vaccine design, longitudinal studies that investigate the co-evolution of HIV-1 and the neutralizing antibody response in single individuals may provide guidance for the design of antigens driving bNAb potency and breadth. For example, several studies revealed that development of broad neutralization was preceded by viral diversification and/or supported by antibody helper lineages that selected for viral variants that drove bNAb development [135,152,154,156,157,158,160,161]. While these observations support a stepwise immunization approach, “dead-end” limbs of antibody lineages appear during the affinity maturation process. Therefore, sequential immunogens will need to be carefully selected [48,135,156,157].

Besides the lineage specific analyses, high-throughput NGS approaches have also been used to investigate the whole B cell receptor repertoire of HIV-1-infected individuals either from combinatorial libraries [162,163,164] or from PBMCs or purified B cells [75,154,165,166,167,168]. A recent study by Waltari et al. detected slight shifts in V gene family usage, higher degrees of somatic hypermutation, and longer CDRH3s for HIV-1-infected individuals [167]. However, other studies could not find any differences but report variation within healthy or HIV-1-infected individuals to be as large as between the different cohorts [75,166]. The current sampling and sequencing depths might therefore still hamper the identification of HIV-1 infection-induced changes on the B cell receptor repertoire.

## 6. Conclusions

Only a small fraction of HIV-1-infected individuals is able to mount a broadly neutralizing serum activity against HIV-1. Over the last decade, advances in screening methods and single cell cloning techniques enabled the isolation of numerous broadly neutralizing antibodies. These antibodies have been shown to be promising candidates for HIV-1 treatment and prevention. However, molecular analyses also revealed special characteristics such as V gene restriction, long CDRH3s, and/or high loads of SHM, which may restrict the development of highly potent bNAbs in natural infection and hamper their induction by current vaccination strategies.

To overcome potential roadblocks for the induction of bNAbs through vaccination, a number of strategies have been proposed. All of these, however, will require the interaction of one or multiple immunogens with B cell receptors to effectively drive bNAb development. Thus, a detailed understanding of the naïve B cell receptor repertoire and the constantly adapting antibody response in the context of HIV-1 infection can be highly informative for vaccine design. Novel experimental and bioinformatics pipelines have the capacity to integrate neutralization, antibody sequence, and structural data. These methods hold great promise to identify common pathways of potent immune responses that will be critical for developing effective vaccination strategies.

## Figures and Tables

**Table 1:** Convergent antibody V gene usage in natural infection and in vaccine trials.

VH	VL	Pathogen	Donor	Reference
IGHV1-2	-	DENV^1^	Acute Dengue Infection	[107]
	-	HIV-1	HIV-1-Infected	[30,72,101,102,104]
IGHV1-3	-	HBV^2^	HBV-Associated Acute Liver Failure Patients	[108]
IGHV1-18	-	Influenza A	Participants of Influenza Vaccine Trial	[32]
	-	DENV	Acute Dengue Infection	[107]
IGHV1-46	-	RV^3^	RV-Infected Adults/Infants	[109,110,111]
	-	HIV-1	HIV-1-Infected	[72,112]
IGHV1-69	-	Influenza A	Hu. Non-Immune Antibody Phage-Display Library	[113]
			Seasonal Influenza Vaccinees	[114]
			Human Donor with Influenza A Broadly Neutralizing Serum	[115]
	-	HIV-1	HIV-1-Infected	[72,101,116]
IGHV3-7	-	HBV	HBV Vaccinees	[117]
	-	Influenza A	Vaccinated Healthy Individuals	[118]
IGHV3-15	IGLV1-40	EBOV^4^	rVSV-ZEBOV^5^ Vaccinees	[34]
			ChAD3 EBOV^6^ Vaccinees	[119]
			Survivor of 2014 EBOV Outbreak in Zaire	[120]
IGHV3-23	IGKV2D-29	Hib^7^	Hib-PS^8^ Conjugate-Vaccinated Infants	[121]
	IGKV1-5	ZIKV^9^	Brazilian/Mexican DENV- and ZIKV-Infected Individuals	[33]
IGHV3-30	IGKV3-11	HCMV^10^	HCMV-Infected Individuals	[122]
		Streptococcus pneumonieae	N/A	[123]
	-	ZIKV	ZIKV-Infected Donors	[124,125]
			Phage-Display Naive Antibody Library	[126]
IGHV4-30-4		RV	RV-Infected Adults/Infants	[109]
IGHV4-1		RV	RV-Infected Adults/Infants	[109]
IGHV4-39		RV	RV-Infected Adults/Infants	[109]
IGHV4-61		RV	RV-Infected Adults/Infants	[109]
IGHV5-51	-	HIV-1	HIV-1-Infected	[127]

^1^ Dengue virus, ^2^ Hepatitis B virus, ^3^ Rotavirus, ^4^ Ebola virus, ^5^ Recombinant vesicular stomatitis virus–Zaire Ebola virus, ^6^ Chimpanzee adenovirus 3 encoding EBOV glycoprotein, ^7^ Haemophilus influenzae type b, ^8^ Haemophilus influenzae type b capsular polysaccharide, ^9^ Zika virus, ^10^ Human cytomegalovirus, VH: V gene segment of the heavy chain, VL: V gene segment of the light chain.
